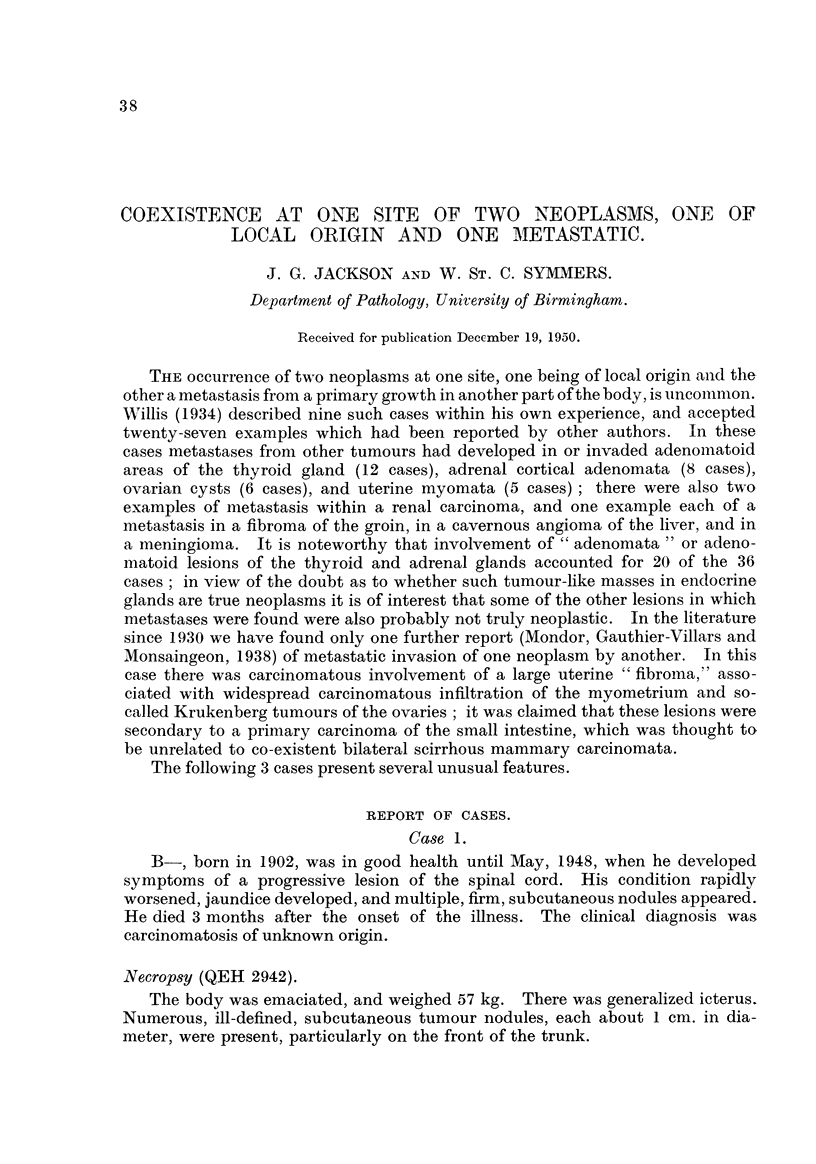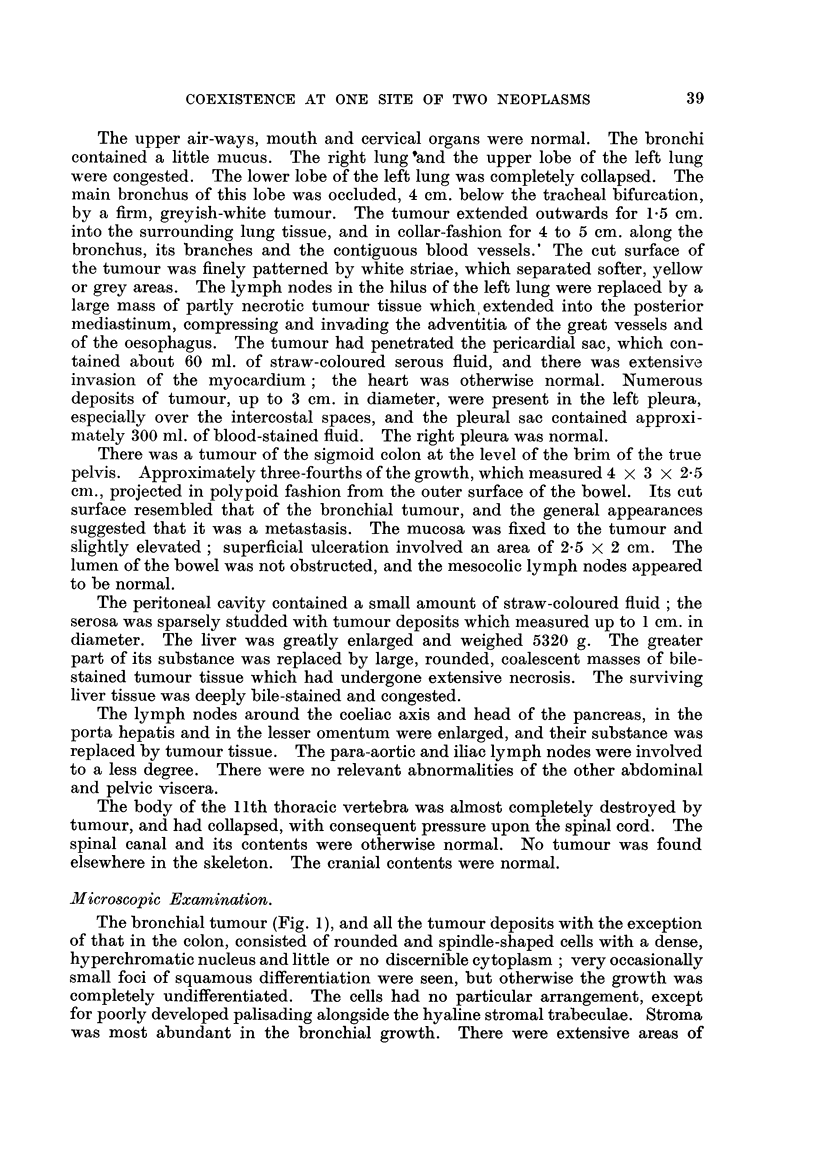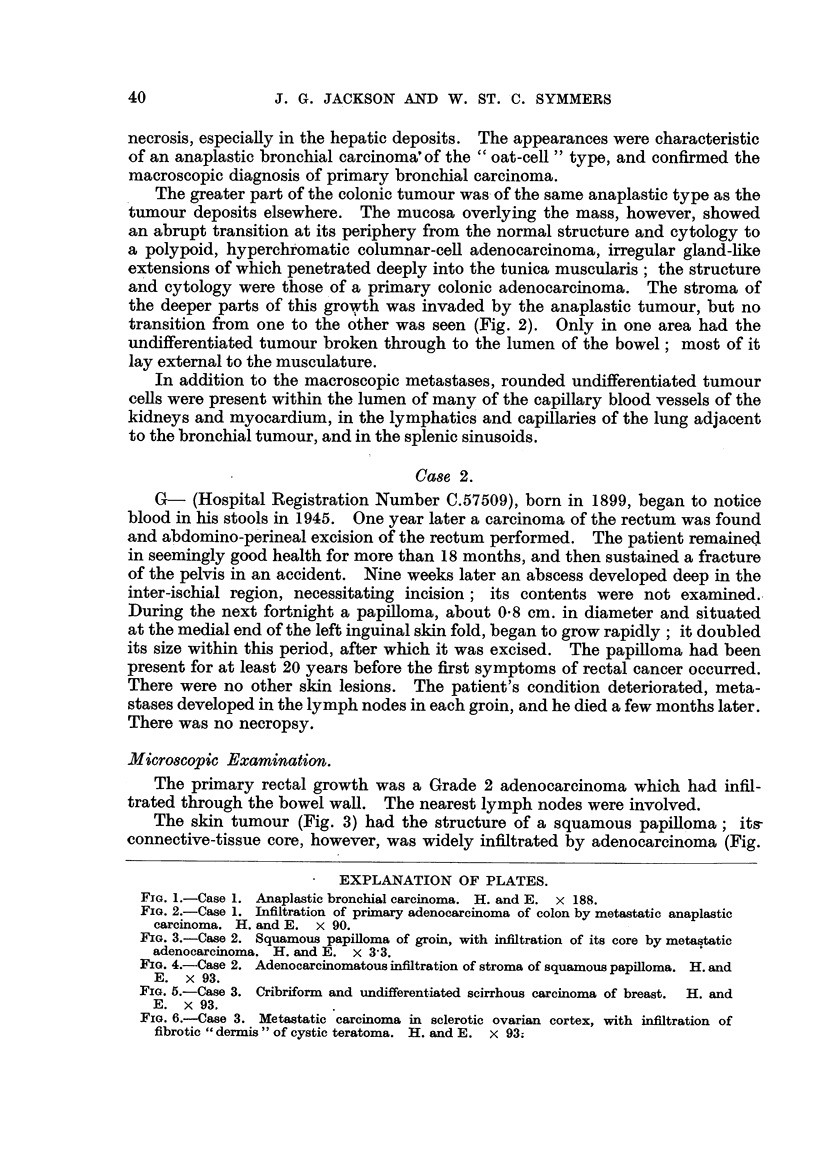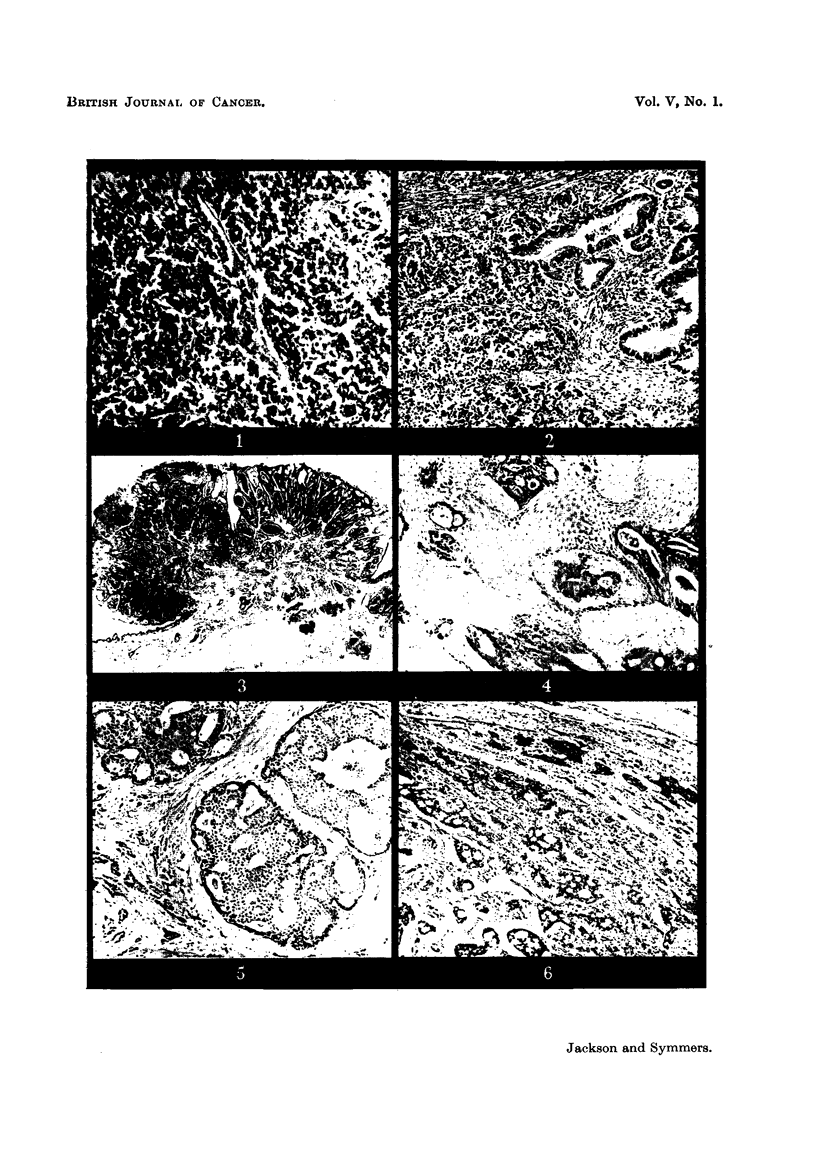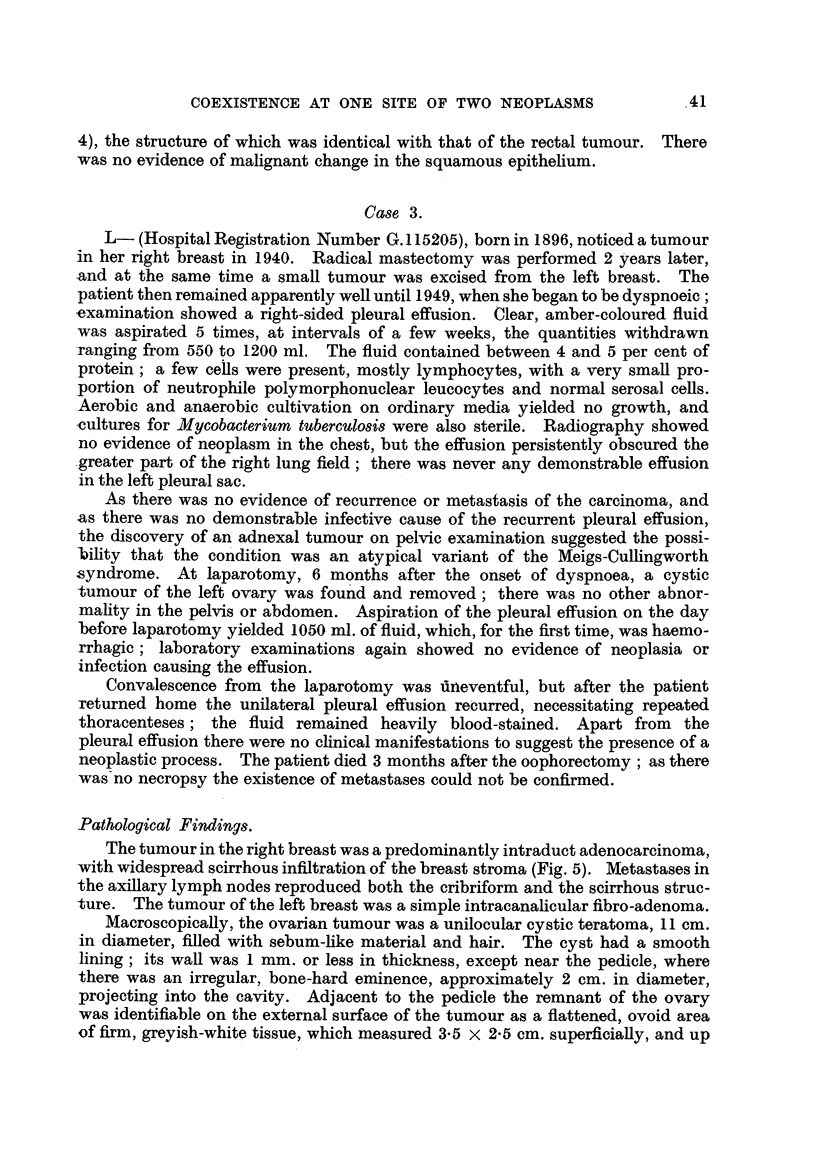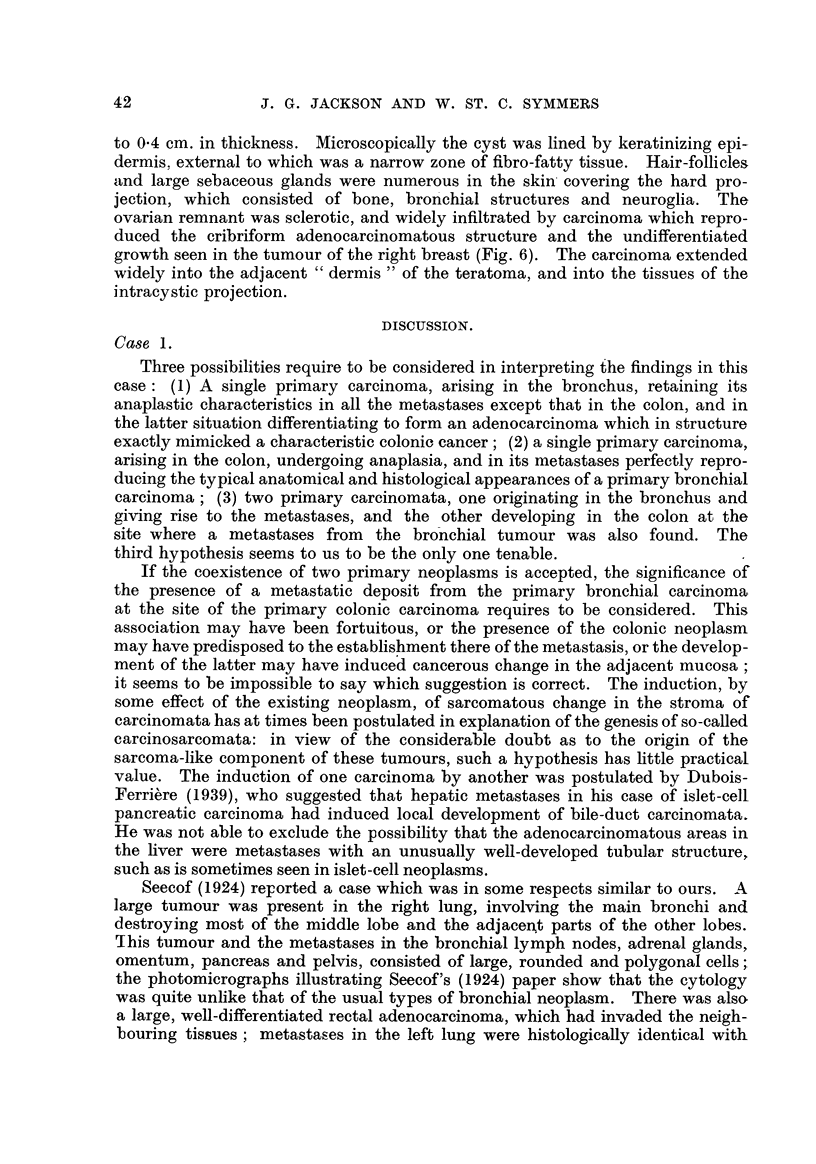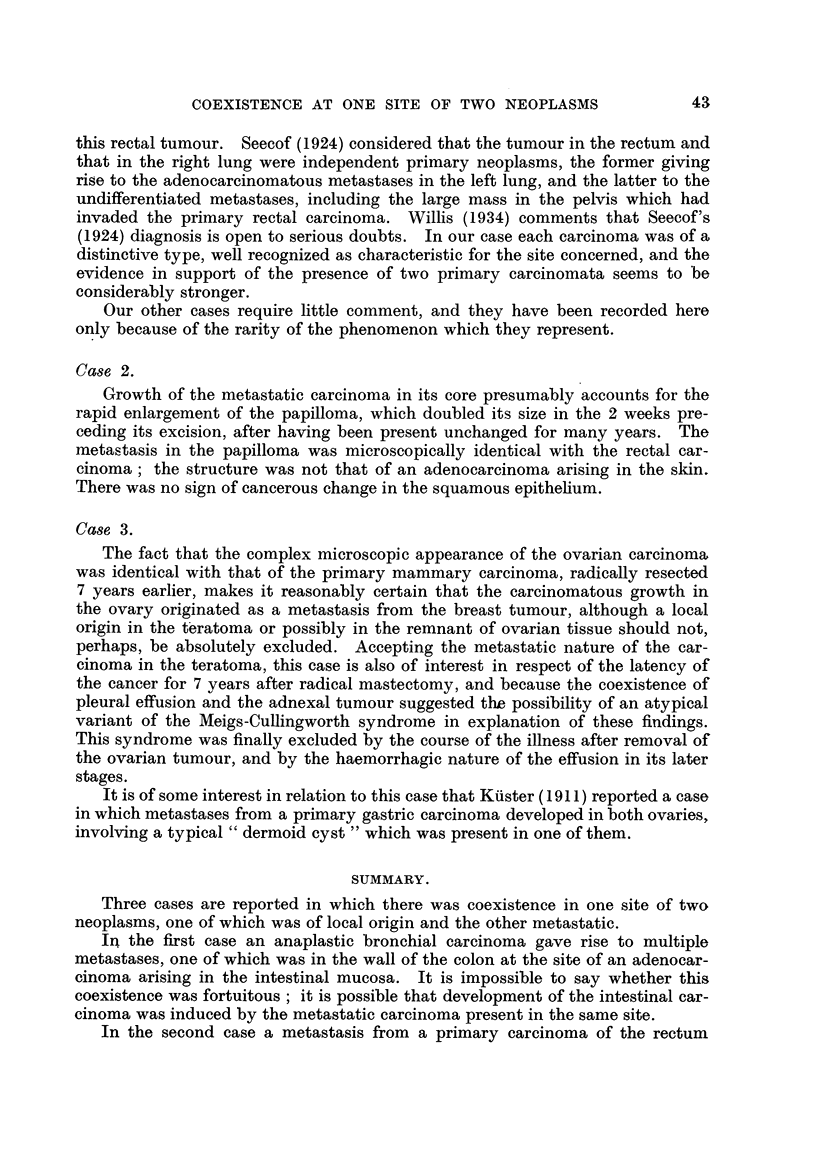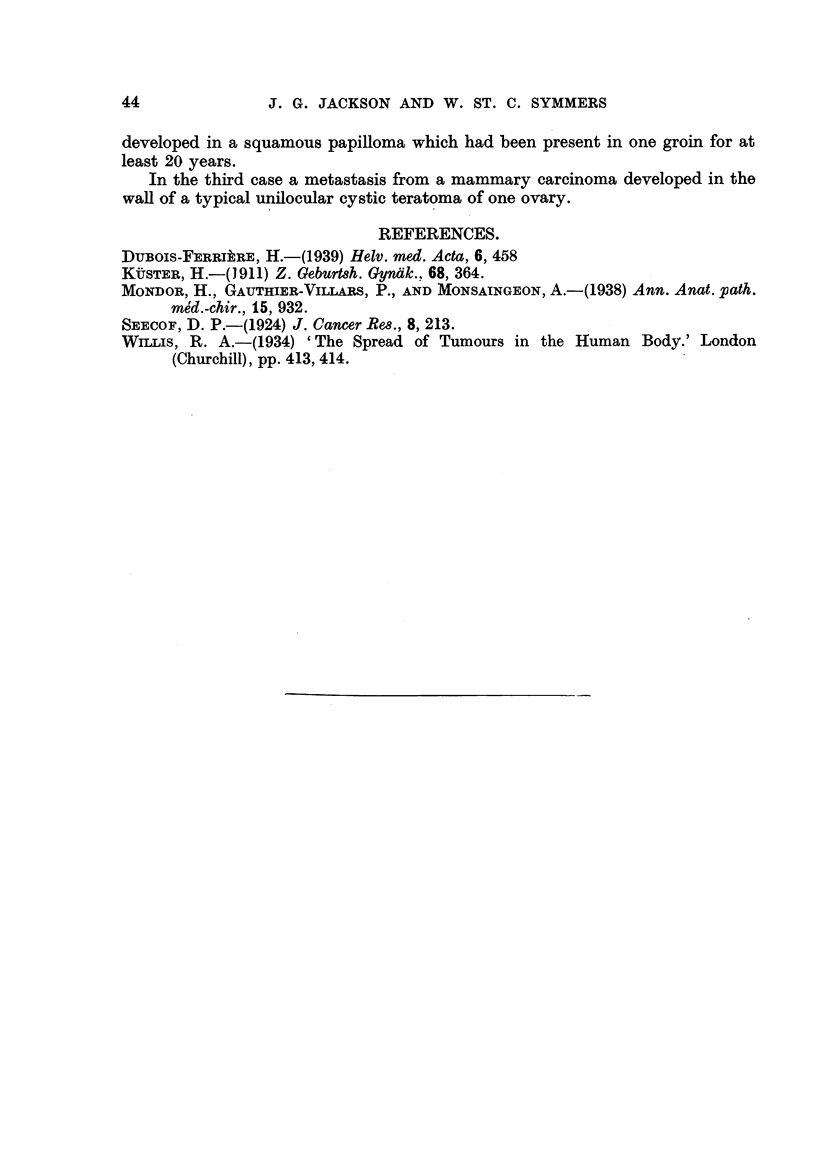# Coexistence at One Site of Two Neoplasms, One of Local Origin and One Metastatic

**DOI:** 10.1038/bjc.1951.4

**Published:** 1951-03

**Authors:** J. G. Jackson, W. St. C. Symmers

## Abstract

**Images:**


### COEXISTENCE AT ONE SITE OF TWO NEOPLASMS, ONE OF LOCAL ORIGIN AND ONE METASTATIC.

J. G. JACKSON AND W. ST. C. SYMMERS.

*Department of Pathology, University of Birmingham.*

Received for publication December 19, 1950.

THE occurrence of two neoplasms at one site, one being of local origin and the other a metastasis from a primary growth in another part of the body, is uncommon. Willis (1934) described nine such cases within his own experience, and accepted twenty-seven examples which had been reported by other authors. In these cases metastases from other tumours had developed in or invaded adenomatoid areas of the thyroid gland (12 cases), adrenal cortical adenomata (8 cases), ovarian cysts (6 cases), and uterine myomata (5 cases) ; there were also two examples of metastasis within a renal carcinoma, and one example each of a metastasis in a fibroma of the groin, in a cavernous angioma of the liver, and in a meningioma. It is noteworthy that involvement of " adenomata " or adenomatoid lesions of the thyroid and adrenal glands accounted for 20 of the 36 cases ; in view of the doubt as to whether such tumour-like masses in endocrine glands are true neoplasms it is of interest that some of the other lesions in which metastases were found were also probably not truly neoplastic. In the literature since 1930 we have found only one further report (Mondor, Gauthier-Villars and Monsaingeon, 1938) of metastatic invasion of one neoplasm by another. In this case there was carcinomatous involvement of a large uterine " fibroma," associated with widespread carcinomatous infiltration of the myometrium and so-called Krukenberg tumours of the ovaries ; it was claimed that these lesions were secondary to a primary carcinoma of the small intestine, which was thought to be unrelated to co-existent bilateral scirrhous mammary carcinomata.

The following 3 cases present several unusual features.

### REPORT OF CASES.

### *Case* 1.

B—, born in 1902, was in good health until May, 1948, when he developed symptoms of a progressive lesion of the spinal cord. His condition rapidly worsened, jaundice developed, and multiple, firm, subcutaneous nodules appeared. He died 3 months after the onset of the illness. The clinical diagnosis was carcinomatosis of unknown origin.

*Necropsy* (QEH 2942).

The body was emaciated, and weighed 57 kg. There was generalized icterus. Numerous, ill-defined, subcutaneous tumour nodules, each about 1 cm. in diameter, were present, particularly on the front of the trunk.

The upper air-ways, mouth and cervical organs were normal. The bronchi contained a little mucus. The right lung and the upper lobe of the left lung were congested. The lower lobe of the left lung was completely collapsed. The main bronchus of this lobe was occluded, 4 cm. below the tracheal bifurcation, by a firm, greyish-white tumour. The tumour extended outwards for 1·5 cm. into the surrounding lung tissue, and in collar-fashion for 4 to 5 cm. along the bronchus, its branches and the contiguous blood vessels. The cut surface of the tumour was finely patterned by white striae, which separated softer, yellow or grey areas. The lymph nodes in the hilus of the left lung were replaced by a large mass of partly necrotic tumour tissue which extended into the posterior mediastinum, compressing and invading the adventitia of the great vessels and of the oesophagus. The tumour had penetrated the pericardial sac, which contained about 60 ml. of straw-coloured serous fluid, and there was extensive invasion of the myocardium ; the heart was otherwise normal. Numerous deposits of tumour, up to 3 cm. in diameter, were present in the left pleura, especially over the intercostal spaces, and the pleural sac contained approximately 300 ml. of blood-stained fluid. The right pleura was normal.

There was a tumour of the sigmoid colon at the level of the brim of the true pelvis. Approximately three-fourths of the growth, which measured 4 × 3 × 2·5 cm., projected in polypoid fashion from the outer surface of the bowel. Its cut surface resembled that of the bronchial tumour, and the general appearances suggested that it was a metastasis. The mucosa was fixed to the tumour and slightly elevated ; superficial ulceration involved an area of 2·5 × 2 cm. The lumen of the bowel was not obstructed, and the mesocolic lymph nodes appeared to be normal.

The peritoneal cavity contained a small amount of straw-coloured fluid ; the serosa was sparsely studded with tumour deposits which measured up to 1 cm. in diameter. The liver was greatly enlarged and weighed 5320 g. The greater part of its substance was replaced by large, rounded, coalescent masses of bile-stained tumour tissue which had undergone extensive necrosis. The surviving liver tissue was deeply bile-stained and congested.

The lymph nodes around the coeliac axis and head of the pancreas, in the porta hepatis and in the lesser omentum were enlarged, and their substance was replaced by tumour tissue. The para-aortic and iliac lymph nodes were involved to a less degree. There were no relevant abnormalities of the other abdominal and pelvic viscera.

The body of the 11th thoracic vertebra was almost completely destroyed by tumour, and had collapsed, with consequent pressure upon the spinal cord. The spinal canal and its contents were otherwise normal. No tumour was found elsewhere in the skeleton. The cranial contents were normal.

*Microscopic Examination.*

The bronchial tumour (Fig. 1), and all the tumour deposits with the exception of that in the colon, consisted of rounded and spindle-shaped cells with a dense, hyperchromatic nucleus and little or no discernible cytoplasm ; very occasionally small foci of squamous differentiation were seen, but otherwise the growth was completely undifferentiated. The cells had no particular arrangement, except for poorly developed palisading alongside the hyaline stromal trabeculae. Stroma was most abundant in the bronchial growth. There were extensive areas of necrosis, especially in the hepatic deposits. The appearances were characteristic of an anaplastic bronchial carcinoma of the "oat-cell" type, and confirmed the macroscopic diagnosis of primary bronchial carcinoma.

The greater part of the colonic tumour was of the same anaplastic type as the tumour deposits elsewhere. The mucosa overlying the mass, however, showed an abrupt transition at its periphery from the normal structure and cytology to a polypoid, hyperchromatic columnar-cell adenocarcinoma, irregular gland-like extensions of which penetrated deeply into the tunica muscularis; the structure and cytology were those of a primary colonic adenocarcinoma. The stroma of the deeper parts of this growth was invaded by the anaplastic tumour, but no transition from one to the other was seen (Fig. 2). Only in one area had the undifferentiated tumour broken through to the lumen of the bowel; most of it lay external to the musculature.

In addition to the macroscopic metastases, rounded undifferentiated tumour cells were present within the lumen of many of the capillary blood vessels of the kidneys and myocardium, in the lymphatics and capillaries of the lung adjacent to the bronchial tumour, and in the splenic sinusoids.

*Case* 2.

G— (Hospital Registration Number C.57509), born in 1899, began to notice blood in his stools in 1945. One year later a carcinoma of the rectum was found and abdomino-perineal excision of the rectum performed. The patient remained in seemingly good health for more than 18 months, and then sustained a fracture of the pelvis in an accident. Nine weeks later an abscess developed deep in the inter-ischial region, necessitating incision; its contents were not examined. During the next fortnight a papilloma, about 0·8 cm. in diameter and situated at the medial end of the left inguinal skin fold, began to grow rapidly; it doubled its size within this period, after which it was excised. The papilloma had been present for at least 20 years before the first symptoms of rectal cancer occurred. There were no other skin lesions. The patient's condition deteriorated, metastases developed in the lymph nodes in each groin, and he died a few months later. There was no necropsy.

*Microscopic Examination.*

The primary rectal growth was a Grade 2 adenocarcinoma which had infiltrated through the bowel wall. The nearest lymph nodes were involved.

The skin tumour (Fig. 3) had the structure of a squamous papilloma; its connective-tissue core, however, was widely infiltrated by adenocarcinoma (Fig.

EXPLANATION OF PLATES.

FIG. 1.—Case 1. Anaplastic bronchial carcinoma. H. and E. × 188.

FIG. 2.—Case 1. Infiltration of primary adenocarcinoma of colon by metastatic anaplastic carcinoma. H. and E. × 90.

FIG. 3.—Case 2. Squamous papilloma of groin, with infiltration of its core by metastatic adenocarcinoma. H. and E. × 3·3.

FIG. 4.—Case 2. Adenocarcinomatous infiltration of stroma of squamous papilloma. H. and E. × 93.

FIG. 5.—Case 3. Cribriform and undifferentiated scirrhous carcinoma of breast. H. and E. × 93.

FIG. 6.—Case 3. Metastatic carcinoma in sclerotic ovarian cortex, with infiltration of fibrotic "dermis" of cystic teratoma. H. and E. × 93.

Jackson and Symmers.

4), the structure of which was identical with that of the rectal tumour. There was no evidence of malignant change in the squamous epithelium.

### *Case* 3.

L— (Hospital Registration Number G.115205), born in 1896, noticed a tumour in her right breast in 1940. Radical mastectomy was performed 2 years later, and at the same time a small tumour was excised from the left breast. The patient then remained apparently well until 1949, when she began to be dyspnoeic; examination showed a right-sided pleural effusion. Clear, amber-coloured fluid was aspirated 5 times, at intervals of a few weeks, the quantities withdrawn ranging from 550 to 1200 ml. The fluid contained between 4 and 5 per cent of protein; a few cells were present, mostly lymphocytes, with a very small proportion of neutrophile polymorphonuclear leucocytes and normal serosal cells. Aerobic and anaerobic cultivation on ordinary media yielded no growth, and cultures for *Mycobacterium tuberculosis* were also sterile. Radiography showed no evidence of neoplasm in the chest, but the effusion persistently obscured the greater part of the right lung field; there was never any demonstrable effusion in the left pleural sac.

As there was no evidence of recurrence or metastasis of the carcinoma, and as there was no demonstrable infective cause of the recurrent pleural effusion, the discovery of an adnexal tumour on pelvic examination suggested the possibility that the condition was an atypical variant of the Meigs-Cullingworth syndrome. At laparotomy, 6 months after the onset of dyspnoea, a cystic tumour of the left ovary was found and removed; there was no other abnormality in the pelvis or abdomen. Aspiration of the pleural effusion on the day before laparotomy yielded 1050 ml. of fluid, which, for the first time, was haemorrhagic; laboratory examinations again showed no evidence of neoplasia or infection causing the effusion.

Convalescence from the laparotomy was uneventful, but after the patient returned home the unilateral pleural effusion recurred, necessitating repeated thoracenteses; the fluid remained heavily blood-stained. Apart from the pleural effusion there were no clinical manifestations to suggest the presence of a neoplastic process. The patient died 3 months after the oophorectomy; as there was no necropsy the existence of metastases could not be confirmed.

#### *Pathological Findings.*

The tumour in the right breast was a predominantly intraduct adenocarcinoma, with widespread scirrhous infiltration of the breast stroma (Fig. 5). Metastases in the axillary lymph nodes reproduced both the cribriform and the scirrhous structure. The tumour of the left breast was a simple intracanalicular fibro-adenoma.

Macroscopically, the ovarian tumour was a unilocular cystic teratoma, 11 cm. in diameter, filled with sebum-like material and hair. The cyst had a smooth lining; its wall was 1 mm. or less in thickness, except near the pedicle, where there was an irregular, bone-hard eminence, approximately 2 cm. in diameter, projecting into the cavity. Adjacent to the pedicle the remnant of the ovary was identifiable on the external surface of the tumour as a flattened, ovoid area of firm, greyish-white tissue, which measured 3·5 × 2·5 cm. superficially, and up to 0·4 cm. in thickness. Microscopically the cyst was lined by keratinizing epidermis, external to which was a narrow zone of fibro-fatty tissue. Hair-follicles and large sebaceous glands were numerous in the skin covering the hard projection, which consisted of bone, bronchial structures and neuroglia. The ovarian remnant was sclerotic, and widely infiltrated by carcinoma which reproduced the cribriform adenocarcinomatous structure and the undifferentiated growth seen in the tumour of the right breast (Fig. 6). The carcinoma extended widely into the adjacent " dermis " of the teratoma, and into the tissues of the intracystic projection.

### DISCUSSION.

*Case* 1.

Three possibilities require to be considered in interpreting the findings in this case : (1) A single primary carcinoma, arising in the bronchus, retaining its anaplastic characteristics in all the metastases except that in the colon, and in the latter situation differentiating to form an adenocarcinoma which in structure exactly mimicked a characteristic colonic cancer ; (2) a single primary carcinoma, arising in the colon, undergoing anaplasia, and in its metastases perfectly reproducing the typical anatomical and histological appearances of a primary bronchial carcinoma ; (3) two primary carcinomata, one originating in the bronchus and giving rise to the metastases, and the other developing in the colon at the site where a metastases from the bronchial tumour was also found. The third hypothesis seems to us to be the only one tenable.

If the coexistence of two primary neoplasms is accepted, the significance of the presence of a metastatic deposit from the primary bronchial carcinoma at the site of the primary colonic carcinoma requires to be considered. This association may have been fortuitous, or the presence of the colonic neoplasm may have predisposed to the establishment there of the metastasis, or the development of the latter may have induced cancerous change in the adjacent mucosa ; it seems to be impossible to say which suggestion is correct. The induction, by some effect of the existing neoplasm, of sarcomatous change in the stroma of carcinomata has at times been postulated in explanation of the genesis of so-called carcinosarcomata: in view of the considerable doubt as to the origin of the sarcoma-like component of these tumours, such a hypothesis has little practical value. The induction of one carcinoma by another was postulated by Dubois-Ferrière (1939), who suggested that hepatic metastases in his case of islet-cell pancreatic carcinoma had induced local development of bile-duct carcinomata. He was not able to exclude the possibility that the adenocarcinomatous areas in the liver were metastases with an unusually well-developed tubular structure, such as is sometimes seen in islet-cell neoplasms.

Seecof (1924) reported a case which was in some respects similar to ours. A large tumour was present in the right lung, involving the main bronchi and destroying most of the middle lobe and the adjacent parts of the other lobes. This tumour and the metastases in the bronchial lymph nodes, adrenal glands, omentum, pancreas and pelvis, consisted of large, rounded and polygonal cells; the photomicrographs illustrating Seecof's (1924) paper show that the cytology was quite unlike that of the usual types of bronchial neoplasm. There was also a large, well-differentiated rectal adenocarcinoma, which had invaded the neighbouring tissues ; metastases in the left lung were histologically identical with this rectal tumour. Seecof (1924) considered that the tumour in the rectum and that in the right lung were independent primary neoplasms, the former giving rise to the adenocarcinomatous metastases in the left lung, and the latter to the undifferentiated metastases, including the large mass in the pelvis which had invaded the primary rectal carcinoma. Willis (1934) comments that Seecof's (1924) diagnosis is open to serious doubts. In our case each carcinoma was of a distinctive type, well recognized as characteristic for the site concerned, and the evidence in support of the presence of two primary carcinomata seems to be considerably stronger.

Our other cases require little comment, and they have been recorded here only because of the rarity of the phenomenon which they represent.

*Case* 2.

Growth of the metastatic carcinoma in its core presumably accounts for the rapid enlargement of the papilloma, which doubled its size in the 2 weeks preceding its excision, after having been present unchanged for many years. The metastasis in the papilloma was microscopically identical with the rectal carcinoma ; the structure was not that of an adenocarcinoma arising in the skin. There was no sign of cancerous change in the squamous epithelium.

*Case* 3.

The fact that the complex microscopic appearance of the ovarian carcinoma was identical with that of the primary mammary carcinoma, radically resected 7 years earlier, makes it reasonably certain that the carcinomatous growth in the ovary originated as a metastasis from the breast tumour, although a local origin in the teratoma or possibly in the remnant of ovarian tissue should not, perhaps, be absolutely excluded. Accepting the metastatic nature of the carcinoma in the teratoma, this case is also of interest in respect of the latency of the cancer for 7 years after radical mastectomy, and because the coexistence of pleural effusion and the adnexal tumour suggested the possibility of an atypical variant of the Meigs-Cullingworth syndrome in explanation of these findings. This syndrome was finally excluded by the course of the illness after removal of the ovarian tumour, and by the haemorrhagic nature of the effusion in its later stages.

It is of some interest in relation to this case that Küster (1911) reported a case in which metastases from a primary gastric carcinoma developed in both ovaries, involving a typical " dermoid cyst " which was present in one of them.

### SUMMARY.

Three cases are reported in which there was coexistence in one site of two neoplasms, one of which was of local origin and the other metastatic.

In the first case an anaplastic bronchial carcinoma gave rise to multiple metastases, one of which was in the wall of the colon at the site of an adenocarcinoma arising in the intestinal mucosa. It is impossible to say whether this coexistence was fortuitous ; it is possible that development of the intestinal carcinoma was induced by the metastatic carcinoma present in the same site.

In the second case a metastasis from a primary carcinoma of the rectum developed in a squamous papilloma which had been present in one groin for at least 20 years.

In the third case a metastasis from a mammary carcinoma developed in the wall of a typical unilocular cystic teratoma of one ovary.

### REFERENCES.

DUBOIS-FERRIÈRE, H.—(1939) *Helv. med. Acta*, **6**, 458

KÜSTER, H.—(1911) *Z. Geburtsh. Gynäk.*, **68**, 364.

MONDOR, H., GAUTHIER-VILLARS, P., AND MONSAINGEON, A.—(1938) *Ann. Anat. path. méd.-chir.*, **15**, 932.

SEECOF, D. P.—(1924) *J. Cancer Res.*, **8**, 213.

WILLIS, R. A.—(1934) 'The Spread of Tumours in the Human Body.' London (Churchill), pp. 413, 414.